# Telomerase Deficiency Predisposes to Heart Failure and Ischemia-Reperfusion Injury

**DOI:** 10.3389/fcvm.2019.00031

**Published:** 2019-04-02

**Authors:** Karima Ait-Aissa, James S. Heisner, Laura E. Norwood Toro, Dennis Bruemmer, Genevieve Doyon, Leanne Harmann, Aron Geurts, Amadou K. S. Camara, Andreas M. Beyer

**Affiliations:** ^1^Cardiovascular Center, Department of Medicine, Medical College of Wisconsin, Milwaukee, WI, United States; ^2^Department of Anesthesiology, Medical College of Wisconsin, Milwaukee, WI, United States; ^3^Vascular Medicine Institute, University of Pittsburgh, Pittsburgh, PA, United States; ^4^Department of Physiology, Medical College of Wisconsin, Milwaukee, WI, United States

**Keywords:** telomerase (TERT), ischema-reperfusion injury, mitochondia, heart disease, reactive oxygen species

## Abstract

**Introduction:** Elevated levels of mitochondrial reactive oxygen species (ROS) contribute to the development of numerous cardiovascular diseases. TERT, the catalytic subunit of telomerase, has been shown to translocate to mitochondria to suppress ROS while promoting ATP production. Acute overexpression of TERT increases survival and decreases infarct size in a mouse model of myocardial infarct, while decreased telomerase activity predisposes to mitochondrial defects and heart failure. In the present study, we examined the role of TERT on cardiac structure and function under basal conditions and conditions of acute or prolonged stress in a novel rat model of TERT deficiency.

**Methods:** Cardiac structure and function were evaluated via transthoracic echocardiogram. Langendorff preparations were used to test the effects of acute global ischemia reperfusion injury on cardiac function and infarction. Coronary flow and left ventricular pressure were measured during and after ischemia/reperfusion (I/R). Mitochondrial DNA integrity was measured by PCR and mitochondrial respiration was assessed in isolated mitochondria using an Oxygraph. Angiotensin II infusion was used as an established model of systemic stress.

**Results:** No structural changes (echocardiogram) or coronary flow/left ventricle pressure (isolated hearts) were observed in TERT^−/−^ rats at baseline; however, after I/R, coronary flow was significantly reduced in TERT^−/−^ compared to wild type (WT) rats, while diastolic Left Ventricle Pressure was significantly elevated (*n* = 6 in each group; *p* < 0.05) in the TERT^−/−^. Interestingly, infarct size was less in TERT^−/−^ rats compared to WT rats, while mitochondrial respiratory control index decreased and mitochondrial DNA lesions increased in TERT^−/−^ compared to WT. Angiotensin II treatment did not alter cardiac structure or function; however, it augmented the infarct size significantly more in TERT^−/−^ compared to the WT.

**Conclusion:** Absence of TERT activity increases susceptibility to stress like cardiac injury. These results suggest a critical role of telomerase in chronic heart disease.

## Introduction

Heart disease is the leading cause of death in the western world and its prevalence continues to increase (1). Chronic inflammation promotes intimal thickening and plaque formation which narrows the vascular lumen and compromises blood flow. With coronary artery disease (CAD), plaque grows within the walls of the coronary arteries until the blood flow to the heart's muscle is limited and causes ischemia (2). Aging is a primary risk factor for development of CAD. Age-related heart morphological changes, functional alterations, and accompanying comorbidities may all contribute to the heart vulnerability during aging (3–5). However, the molecular and genetic mechanisms related to aging induced vascular changes are poorly understood.

Telomere length and telomerase activity are best known for their role in cellular aging. Decreased Telomere length has been associated with CAD (2); however, recent evidence points toward a telomere-independent role of telomerase in cardiovascular disease (6, 7). The canonical role of TERT, the catalytic subunit of telomerase, is to elongate chromosomal ends (telomeres) during cell division (8). A non-canonical role of TERT in preventing increased reactive oxygen species (ROS) and reduced ATP generation due to increased mitochondrial DNA (_mt_DNA) mutations has been established (9–12). We recently showed that in atrial vessels from human subjects with CAD, the increase in mitochondrial ROS (_mt_ROS) altered endothelial function and reduced the bioavailability of nitric oxide (13, 14); the increase in TERT can modulate cellular redox state by upregulation of mitochondrial antioxidant enzymes (15), reduce _mt_ROS production (16) and reverse the impaired phenotype observed in coronary vessels from CAD human subjects (6).

_mt_DNA damage and changes in cardiac metabolism have been linked to the development of cardiomyopathy (17) and heart failure (18). The concomitant dysregulation of _mt_DNA may harm respiratory chain subunits thereby creating a feed-forward loop of further oxidative stress and bioenergetics failure, two features implicated in the mitochondrial theory of aging (19). This theory is supported by the findings that an increase in _mt_DNA damage increases _mt_ROS levels, while decreasing mitochondrial metabolism. Additionally, the decline in electron transport chain activity, which is often associated with impaired ATP generation, has been shown to be implicated in cardiac hypertrophy and HF (20–22). Interestingly, emerging evidence showed that following an external stress, TERT reversibly translocated from the nucleus to the mitochondria, where it exerted a protective role by binding to _mt_DNA, suppressing _mt_ROS production (23–27) and increasing respiratory chain activity (27). Cardiac specific induced TERT overexpression in a myocardial infarction mouse model has been shown to attenuate cardiac dilation, improve ventricular function and decrease infarct size in left anterior descending artery ligation (7), and decrease apoptosis *in vivo* and in cultured cardiomyocytes (28). Conversely, decreased TERT activity potentiates mitochondrial and cellular oxidative stress (23, 24) and promotes impaired cell division, enhanced cardiomyocyte hypertrophy and death, which are associated with ventricular dilation, thinning of the ventricular wall and cardiac dysfunction (29). Our recently published work shows that TERT^−/−^ mice are pre-disposed to endothelial dysfunction induced by Ang II, while TERT overexpressed mice were protected from this effect (30). As the renin angiotensin system is a well characterized contributor to cardiac damage, including remodeling and heart failure (31, 32), we used Angiotensin II (Ang II) as a systemic stressor to explore the predisposition of TERT knockout rats to external stressors.

We hypothesized that TERT deficiency augments cardiac dysfunction and exacerbate I/R injury. In this context, we examined the effect of TERT deficiency on cardiac structure, function and metabolism under baseline conditions and following ischemic stress in a novel rat model of TERT deficiency.

## Materials and Methods

### Animals

All protocols were approved by the Institutional Animal Care and Use Committee (IACUC) of the Medical College of Wisconsin and conformed to the Guide for the Care and Use of Laboratory Animals published by the National Institutes of Health. Wistar Kyoto (WKY), wild type (WT) littermates (15–20 weeks-old) and TERT knockout rats (TERT^−/−^) (15–20 weeks-old) were generated by Dr. Geurts Laboratory at the Medical College of Wisconsin (33). All rats were housed in groups of five (or less at their adult age), maintained at a temperature of 23°C with 12 h light/dark cycles and fed a solid standard diet (Na^+^ content 0.4%) and water.

### Development of WKY TERT (TERT^−/−^) Knockout Rats

Knockouts for TERT, the catalytic subunit of telomerase, were developed on the inbred Wistar Kyoto (WKY/NCrl) background using CRISPR/Cas9 technology (34, 35). A CRISPR guide RNA targeting the first exon sequence GGGCAACGAGGAGCGCGGGG of *Tert* (protospacer adjacent motif underlined) was successfully used to generate a 17-bp frame shift mutation following pronuclear injection into WKY/NCrl rat embryos, resulting in a premature stop codon and effectively eliminating functional TERT expression. A heterozygous breeding colony of WKY-TERT (WKY-*Tert*^*em*2*Mcwi*^) animals was established and homozygous TERT mutant rats are viable and are born at Mendelian frequencies. Supplemental Figure 2 shows sequence confirmation of the TERT knockout strain and genotyping analysis profile for each strain.

### Telomerase Activity—Telomeric Repeat Amplification Protocol Assay

Telomerase activity was analyzed using a commercially available polymerase chain reaction (PCR)-based assay according to the manufacturer's instructions (TeloTAGGG Telomerase PCR ELISA Plus; Roche Applied Sciences) and as described previously (36). Briefly, spleen samples of 50–60 mg were harvested from rats and homogenized in lysis buffer. After centrifugation, the supernatant containing the whole-cell proteins were quantified using the BioDrop and processed for elongation/amplification PCR. The relative activity was quantified using a generated standard curve.

### Telomere Length

Genomic DNA were obtained from hearts of WT and TERT^−/−^ rats and prepared using E.Z.N.A MicroElute Genomic DNA kit (Promega Bio-tek Inc.) according to the manufacturer's instructions. PCR was performed under the following conditions: 0.75 SYBR Green I (Invitrogen), 10 mM Tris–HCl pH 8.3, 50 mM KCl, 3 mmol/L MgCl2, 0.2 mmol/L each dNTP (deoxynucleotides), 1 mM DTT (dithiothreitol) and 1 M betaine (US Biochemicals). For 25 mL reaction, 0.625U AmpliTaq Gold DNA polymerase (Applied Biosystems, Inc.) was used. Multiplex QPCR primer pair final concentrations were 900 nM each. PCR was performed as follows: step 1 (1X): 95°C for 15 min; Step 2 (36X): 98°C for 2 s, 48°C for 1 min, 74°C for 15 s, 84°C for 30 s, 85°C for 15 s as previously described by Morgan et al. (37).

### Immunohistochemistry

Heart tissues were processed for immunohistochemistry at the Children's Hospital of Wisconsin pathology core as previously described (38, 39). Briefly, tissues were fixed in 10% neutral-buffered formalin, processed, embedded into paraffin blocks and prepared into slides. The TERT antibody (bs-1411R, Bioss, dilution 1:100) was detected and visualized using Bond Polymer Refine Detection System (DS9800) with the addition of a DAB enhancer (AR9432), using the MOD F protocol/software. Omission of the primary antibody served as negative control.

### Assessment of Cardiac Function by Echocardiography

TERT^−/−^ rats (6 males and 7 females) and their counterpart littermates, WT (6 males and 6 females), were subjected to noninvasive two-dimensional echocardiography as previously described (40, 41). Briefly, on the day of the experiment, rats were anesthetized (1–2% isoflurane), and baseline echocardiograms were recorded using a General Electric Vivid 7 system (Waukesha, WI) equipped with an 11 MHz M12L linear transducer. Standard parasternal short axis images were obtained at the mid-left ventricular level (papillary muscles served as markers) by two-dimensional echocardiography. The images were then analyzed using Echo-PAC workstation with Q analysis software (General Electric, Waukesha, WI). LV dimensions were measured in diastole and systole, as well as the anterior and posterior wall thickness in diastole. Fractional shortening (FS), left ventricular mass (LVM) and ejection fraction (EF) were determined using previously described calculations (40, 41). Investigators were blinded to the experimental groups for the duration of the echocardiographic measurements.

### Systemic Cardiovascular Stress

Prolonged systemic stress was induced by promoting a pro-oxidative environment via Ang II infusion (400 ng/kg/min, osmotic mini pump 14 days) (42–45). Control rats were infused with saline for 14 days.

### Langendorff Heart Preparation

The Langendorff procedure used in this study is similar to those we have used previously (46–48). Animals were anesthetized by intraperitoneal injection of 30 mg ketamine along with 1000U heparin and euthanized by decapitation after no response to a noxious stimulus. After thoracotomy, the aorta was cannulated distal to the aortic valve, and the heart perfused retrograde with 4°C oxygenated Krebs Ringers (KR) solution of the following composition (in mM): 148 Na^+^, 4.7 K^+^, 1.2 Mg^2+^, 1.6 Ca^2+^, 127 Cl^−^, 27.8 ${\text{HCO}}_{3}^{-}$, 1.2 ${\text{H}}_{2}{\text{PO}}_{4}^{-}$, 1.2 ${\text{SO}}_{4}^{2-}$, 5.5 glucose, 2 pyruvate, 0.026 EDTA, and 5 U/l insulin. After ligation of the superior and inferior venae cavae, the heart was rapidly harvested and mounted in the Langendorff apparatus and perfused at constant pressure of 70 mmHg at 37°C. The pH was maintained at 7.40 by perfusion with ~95% O_2_ and ~5% CO_2_. Systolic and diastolic left ventricular pressures (LVSP and LVDP respectively) were measured isovolumetrically with a saline-filled latex balloon (Radnoti, Monrovia, CA) inserted into the left ventricle via the left atrium. The volume of the balloon was initially adjusted to achieve a diastolic LVP of 0 mmHg to reflect any subsequent increase in diastolic contracture, an index of I/R injury. Developed LVP (LVSP-LVDP; Dev-LVP) was derived. Spontaneous heart rate (HR) was monitored via electrodes placed on the right atrial and ventricular free walls. The rate pressure product (RPP), an index of workload, was derived from Dev-LVP and HR. Coronary flow (CF) was measured by an ultrasonic flowmeter (Transonic T106X; Ithaca, NY) placed directly in the aortic inflow line. The ionic conditions of the coronary inflow (Krebs Ringer) and outflow of Na^+^, K^+^, Ca^2+^, pCO_2_, and pH were monitored off-line with an intermittently self-calibrating analyzer system (Radiometer Copenhagen ABL 505; Copenhagen, Denmark).

### Experimental Groups and Protocol

The rats were divided into 4 groups: (1) TERT^−/−^ without Ang II (*N* = 8; 3 males and 5 females), (2) WT without Ang II (*N* = 7; 3 males and 4 females), (3) TERT ^−/−^ with Ang II (*N* = 4; 2 males and 2 females), (4) WT with Ang II (*N* = 6, 2 males and 4 females). Hearts from both TERT^−/−^ rats and WT littermates with or without Ang II treatment were allowed to stabilize for 20 min followed by baseline measurements. The hearts were then subjected to 25 min global no-flow ischemia and 120 min reperfusion. At the last 20 min reperfusion, vascular reactivity was determined using Na^+^-nitroproside (SNP), bradykinin (BK) and adenosine (Ade). Time matched non-ischemic controls or time controls (TC; *N* = 6/group) were perfused for the duration of the I/R protocol length of 185 min. Functional variable and mitochondrial redox state (NADH) were recorded online, before, during and after I/R and in TC.

### Spectrofluorometric Measurements of Mitochondrial NADH in the Intact Beating Heart

**Figure 1:**
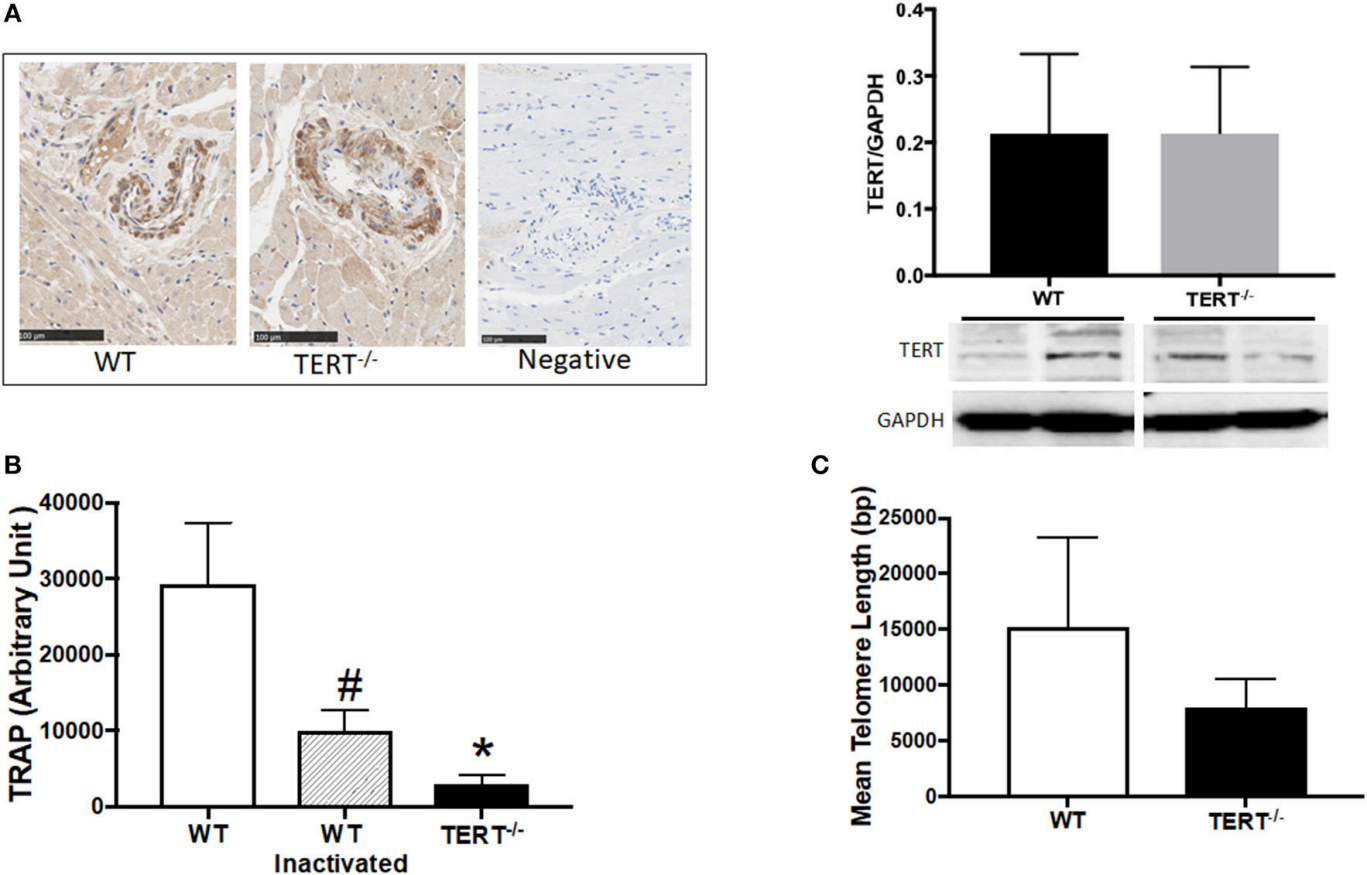
TERT expression and activity assessment in the TERT^−/−^ and WT. **(A)** Immunohistochemistry (left panel) and western blot analysis (right panel) of TERT protein expression in heart sections from TERT^−/−^ compared to WT and no-antibody staining as a negative control for IHC; **(B)** Telomeric repeat amplification protocol (TRAP) assay in tissue lysates from TERT^−/−^, WT, and WT heat inactivated samples; **(C)** Telomere length measurements in hearts from TERT^−/−^ and WT rats. Values are expressed as means ± SEM. #*P* < 0.05 for WT inactivated vs. WT group and *P < 0.05 for TERT^−/−^ vs. WT group.

Cardiac mitochondrial NADH autofluorescence (AF) was measured in the *ex vivo* beating heart using fluorescence technique at λ_ex_ 350 nm and λ_em_ ratio 450 nm/390 nm (Horiba Photon Technology London, Canada) as described before (47, 48). Experiments were conducted in a light-proof Faraday cage to block all incident lights and minimize photo bleaching of fluorescent signals. The signals were acquired by placing a fiber optic probe gently against the LV free wall and connected to the spectro-fluorometer (Figure 1A). The placement of the probe against the heart did not impede the normal cardiac function. Each signal was digitized and recorded at 200 Hz on computers for later signal analyses. The signal intensities were quantified in arbitrary fluorescence unit (a.f.u.).

### Infarct Size Measurement

After 120 min reperfusion, hearts were removed and atria discarded; ventricles were cut into 2 mm transverse sections with a heart matrix and incubated in 1% 2,3,5-triphenyltetrazolium chloride (TTC) in 0.1 M KH_2_PO_4_ buffer (pH 7.4, 38°C) for 10 min (49). TTC stains viable tissue red, indicating the presence of a formazan precipitate that results from TTC reduction by dehydrogenase enzymes present in viable tissue. All slices were digitally imaged on green background by a photo-scanner, and the infarcted areas of each slice analyzed by planimetry using Image J 1.44i software (NIH, Bethesda, MD), with ColorThreshold plug-in, and a recently developed and calibrated macro ensuring fast and operator-independent measurements (49). Infarcted areas of individual slices were averaged to calculate the total infarction of both ventricles.

### Mitochondrial Isolation

Mitochondria were isolated as described previously by differential centrifugation (50). Harvested hearts were immediately immersed in 4°C cold isolation buffer containing in mM: 200 mannitol, 50 sucrose, 5 KH_2_PO_4_, 5 3-(N-morpholino) propane sulfonic acid, and 1 EGTA, with 0.1% bovine serum albumin, pH 7.15, and minced into 1 mm^3^ pieces. All isolation procedures were conducted at 4°C. The tissue was homogenized for 15 s in 2.5 ml isolation buffer containing 5 U/ml protease, and for another 15 s after adding 17 ml isolation buffer. The suspension was centrifuged at 8,000 *g*; the resulting pellet was then re-suspended in isolation buffer and centrifuged again at 800 *g* to remove and discard the cellular debris. The supernatant containing the mitochondrial fraction was further centrifuged at 8,000 *g* and the final mitochondria pellet was suspended in isolation buffer and kept on ice for studies. Total protein concentration was determined by the Bradford method with BSA as a standard.

### Mitochondrial Respiration

Mitochondria (0.25 mg/ml) were suspended in 500 μl experimental buffer (in mM): 130 KCl, 5 K_2_HPO_4_, 20 MOPS, and 2.5 EGTA; 1 μM Na_4_P_2_O_7_, and 0.1% BSA, pH 7.15. O_2_ consumption was measured polygraphically with a Clark-type O_2_ electrode (model 1302; Strathkelvin Instruments, Glasgow, Scotland) as described before (50). To determine the coupling of oxidative phosphorylation, respiratory control index (RCI) was determined as the ratio of the maximum O_2_ consumption rate (state 3) after addition of pyruvate/malate (10 mM) or succinate (10 mM) and ADP (250 μM) to the O_2_ consumption rate during state 4 respiration after complete phosphorylation of ADP to ATP.

### Mitochondrial DNA Damage Analysis:

Quantitative PCR (QPCR) was used to assay _mt_DNA damage as described previously (51). Briefly, total genomic DNA was isolated using QIAGEN Genomic Tip and Genomic DNA Buffer Set Kit (QIAGEN, Valencia, CA). Purified genomic DNA was quantified fluorometrically using Pico Green dsDNA reagent (Molecular Probes, Life Technologies, USA). Lambda (λ)/HindDIII DNA (Gibco Invitrogen, Paisley, UK) was used to generate a standard curve and adjust the final DNA concentration to 3 ng/μL. The “hot start” PCR used the Gene Amp XL PCR Kit (Applied Biosystems, Foster City, CA, USA) with 15 ng DNA, 1X buffer, 100 ng/μL BSA, 200 μM dNTPs, 20 pmol of each primer (Include a Table), 1.3 mM Mg^2+^ and H_2_O to 45 μL. The reaction was brought to 75°C before adding 1 U/reaction enzyme (0.5 μL of polymerase in 4.5 μL H_2_O). Specific primers were used to amplify a large fragment of _mt_DNA (8.9 kb) to determine _mt_DNA integrity; and a small fragment (139 bp) of the mitochondrial genome to monitor changes in _mt_DNA copy number and to normalize the data obtained when amplifying the 8.9-kb fragment. Relative amplifications were calculated to compare KO hearts to WT hearts; these values were used to estimate quantitatively the number of lesions present in DNA, assuming a Poisson distribution as previously described (51).

### Statistical Analysis

Data are presented as means ± SEM. Differences between were determined using a Student's *t*-test and One-Way ANOVA. A probability value of *p* < 0.05 was considered to be statistically significant. Statistical analyses were performed using Graphpad Prism version 7 software.

## Results

### Validation of TERT Deficiency in the TERT^−/−^ Rats and Impact on Telomeric Activity

To confirm the absence of TERT expression, we performed immunohistochemistry (IHC) against TERT protein in paraffin embedded heart sections. Surprisingly, expression of TERT was detected by IHC and western blot in the hearts from both TERT^−/−^ genotyped and WT rats (Figure 1A; *N* = 4 for each group). This indicates that a variant form of TERT was likely generated after CRISPR.

To test whether this variant form of TERT in the KO strain was functional (catalytic active), telomerase activity was evaluated. Figure 1B shows the results obtained using TRAP assay. TERT^−/−^ rats showed marked decrease in telomerase activity compared to WT rats. Denatured WT telomerase (via heat inactivation, HI) was used as a control for specificity of the assay. (*N* = 4 for each group, ^*^p < 0.05 for TERT^−/−^ vs. WT rats, #*p* < 0.05 for WT HI vs. WT).

To further confirm the loss of function of TERT in the KO, we measured their telomere length using a qPCR-based assay (37, 52, 53). Following the loss of the telomerase activity (Figure 1C), the TERT^−/−^ display significantly shorter telomeres compared to their control WT counterparts; however, critical short telomeres (< 1,000 bp) were not observed (*N* = 4 for each group; *p* < 0.05 for TERT^−/−^ vs. WT rats).

### Loss of Telomerase Activity Affect Cardiac Function in Isolated Hearts After Ang II Treatment

Table 1 shows baseline characteristics of TERT^−/−^ and WT with Ang II treated and untreated rats. The TERT^−/−^ rats display similar body and heart weights compared to their corresponding WT controls.

**Table 1:** Summary of baseline and after Ang II infusion on cardiac characteristics of TERT^−/−^ and WT rats.

	**Vehicle**			**AngII**		
	**WT (*N* = 7)**	**TERT^**−/−**^ (*N* = 8)**	***P* value (TERT^**−/−**^ vs. WT)**	**WT (*N* = 6)**	**TERT^**−/−**^ (*N* = 4)**	***P* value (TERT^**−/−**^ vs. WT)**
Body weight (g)	332.1 ± 18.2	308.5 ± 24.4	ns	212 ± 18.3	258.3 ± 31.8	ns
Heart weight (g)	1.38 ± 0.1	1.3 ± 0.1	ns	0.96 ± 0.06	1.09 ± 0.1	ns
Heart rate (beat/min)	186.4 ± 18.7	211.5 ± 19.6	ns	234.1 ± 4.3	147.8 ± 29	0.03
Coronary flow (ml/min)	9.5 ± 1.1	10.7 ± 0.5	ns	7.8 ± 0.9	6.32 ± 1.40	ns
Developed LV pressure (mmHg)	68.2 ± 9.2	75.1 ± 11.4	ns	152.8 ± 7.05	120 ± 8.8	0.02
Rate pressure product (mmHg × beat/min)	11900.8 ± 2512.9	14860.3 ± 3219.4	ns	31858.0 ± 1244.9	14100.0 ± 3969.9	0.0009

Parameters of cardiac function from untreated rats were evaluated by echocardiogram in *in vivo* did not show any difference between TERT^−/−^ and WT rats (Supplemental Figure 1). Basal heart function of the isolated perfused heart, assessed as HR, CF, Dev-LVP, and RPP, was also not different between the knockout and WT group (Table 1). Given these results, we can only speculate that a genetic compensation (54) might have occurred in the KO rats, where at baseline, there was no significant effect of TERT KO in the Ang II untreated rats. To further determine an effect of TERT KO on cardiac function, we evaluated the impact of cardiovascular stressor, Ang II (55), on basal cardiovascular function and the susceptibly to I/R injury.

Although, no changes were observed from the echocardiogram in TERT^−/−^ rats compared to WT, the treatment with Ang II induced a significant decrease in HR, Dev-LVP and RPP in the TERT^−/−^ rats with Ang II (HR: WT: 234.1 ± 4.3 beat/min, *N* = 6 vs. TERT^−/−^: 147.8± 29^*^ beat/min, *N* = 4; dev-LVP: WT: 152.8 ± 7.05 mmHg, *N* = 6 vs. TERT^−/−^: 120 ± 8.8^*^ mmHg, N = 4; RPP: WT: 31858.0 ± 1244.9 mmHg × beat/min, *N* = 6 vs. TERT^−/−^: 14100.0 ± 3969.9^*^ mmHg × beat/min, *N* = 4).

### Lack of Telomerase Activity Hampers Recovery From Ischemia Reperfusion Injury

**Figure 2:**
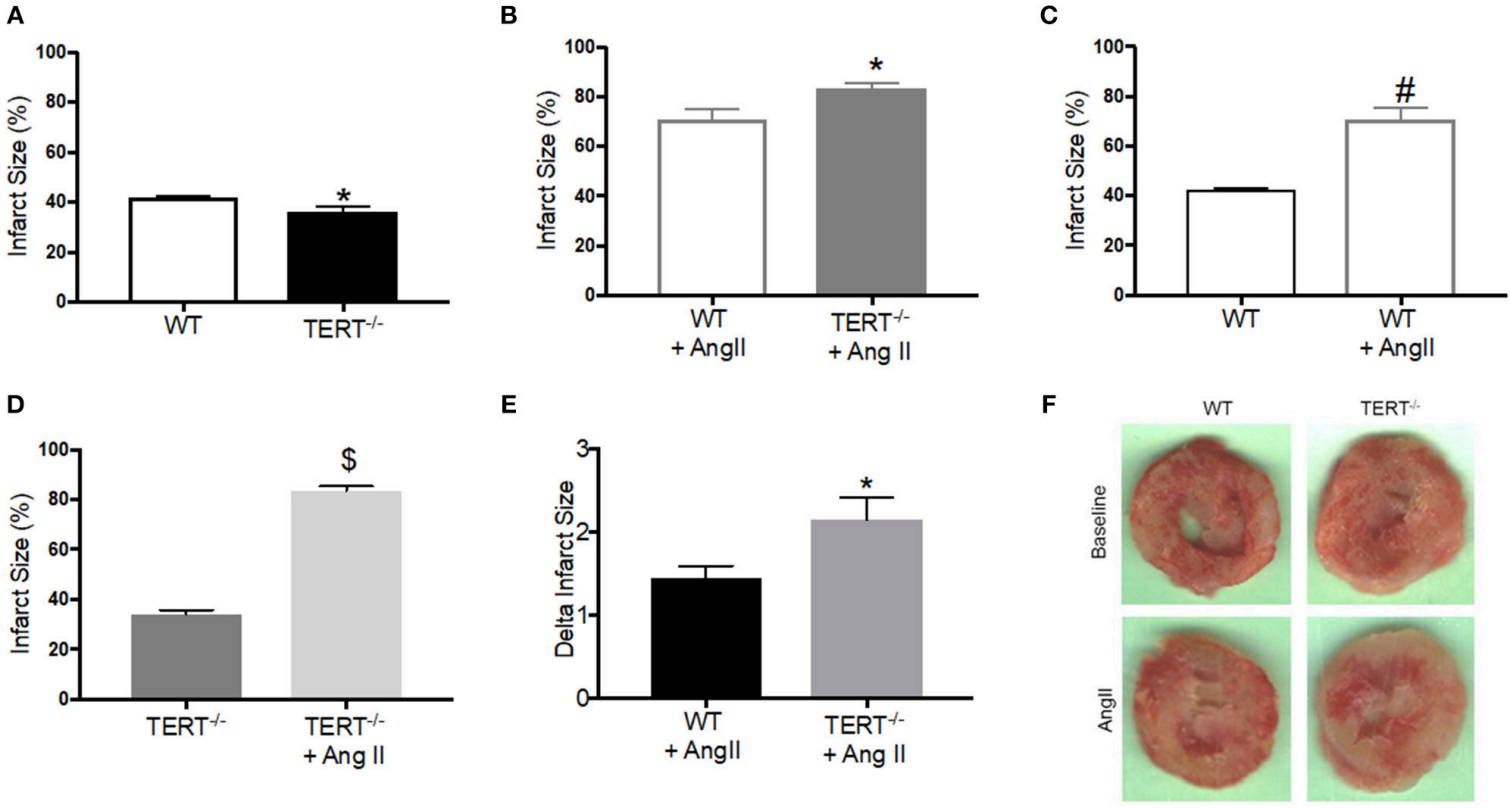
Myocardial Infarct size measured in the TERT^−/−^ and WT after global ischemia in rats with and without Ang II treatment. Infarct size expressed as percent of the area at risk (whole ventricle) in TERT^−/−^ vs. WT without Ang II treatment **(A)**, in TERT^−/−^ vs. WT with Ang II treatment **(B)**, in WT with Ang II vs. WT without Ang II treatment **(C)**, in TERT^−/−^ + Ang II vs. TERT^−/−^ without Ang II treatment **(D)**, delta infarct size for TERT^−/−^ + Ang II vs. WT + Ang II relative to II values for each group **(E)** and representative images of infarct scars after global ischemia in TERT^−/−^ and WT rats infused without (TERT^−/−^: *n* = 3 males and 5 females and WT: *n* = 3 males and 4 females) or with Ang II (TERT^−/−^: *n* = 2 males and 2 females and WT: *n* = 2 males and 4 females) **(F)**. Values are expressed as means ± SEM. *P = 0.04 for TERT^−/−^ +AngII vs. WT+ Ang II; #*P* = 0.0001 for WT +AngII vs. WT; $*P* = 0.0001 for TERT^−/−^ +AngII vs. TERT^−/−^.

We showed previously that CAD subjects display a decrease TERT expression and activity, and its pharmacological inactivation induces a CAD phenotype in healthy human tissues (6). To evaluate the direct impact of decreased telomerase activity *in vivo*, we used a genetic model of TERT ablation. In order to explore whether absence of TERT activity impacts susceptibility to I/R injury, TERT^−/−^ and WT isolated *ex vivo* hearts were subjected to 25 min of no flow global ischemia followed by 120 min reperfusion. Figure 2 shows infarct size of TERT ^−/−^ and WT rat hearts with or without Ang II treatment. Surprisingly, TERT^−/−^ displayed significantly smaller infarct size compared to their WT counterparts in the Ang II untreated rats (Figure 2A; WT: 41.7 ± 0.9%, *n* = 7; TERT^−/−^: 36.3 ± 1.9^*^%, *n* = 8; ^*^p < 0.05 for TERT^−/−^ vs. WT). However, rats treated with Ang II showed significantly greater infarct size in both WT (Figure 2C; *P* = 0.0001) and TERT^−/−^ (Figure 2D; *P* = 0.0001) when compared to the Ang II untreated group; in addition, the TERT^−/−^ displayed a further increase in infarct size compared to the WT hearts (Figures 2B,E; WT: 76.67 ± 4.88%, *n* = 6; TERT^−/−^: 83.3 ± 2.1%, *N* = 4; ^*^P = 0.04 for TERT^−/−^ + Ang II vs. WT+ Ang II).

**Figure 3:**
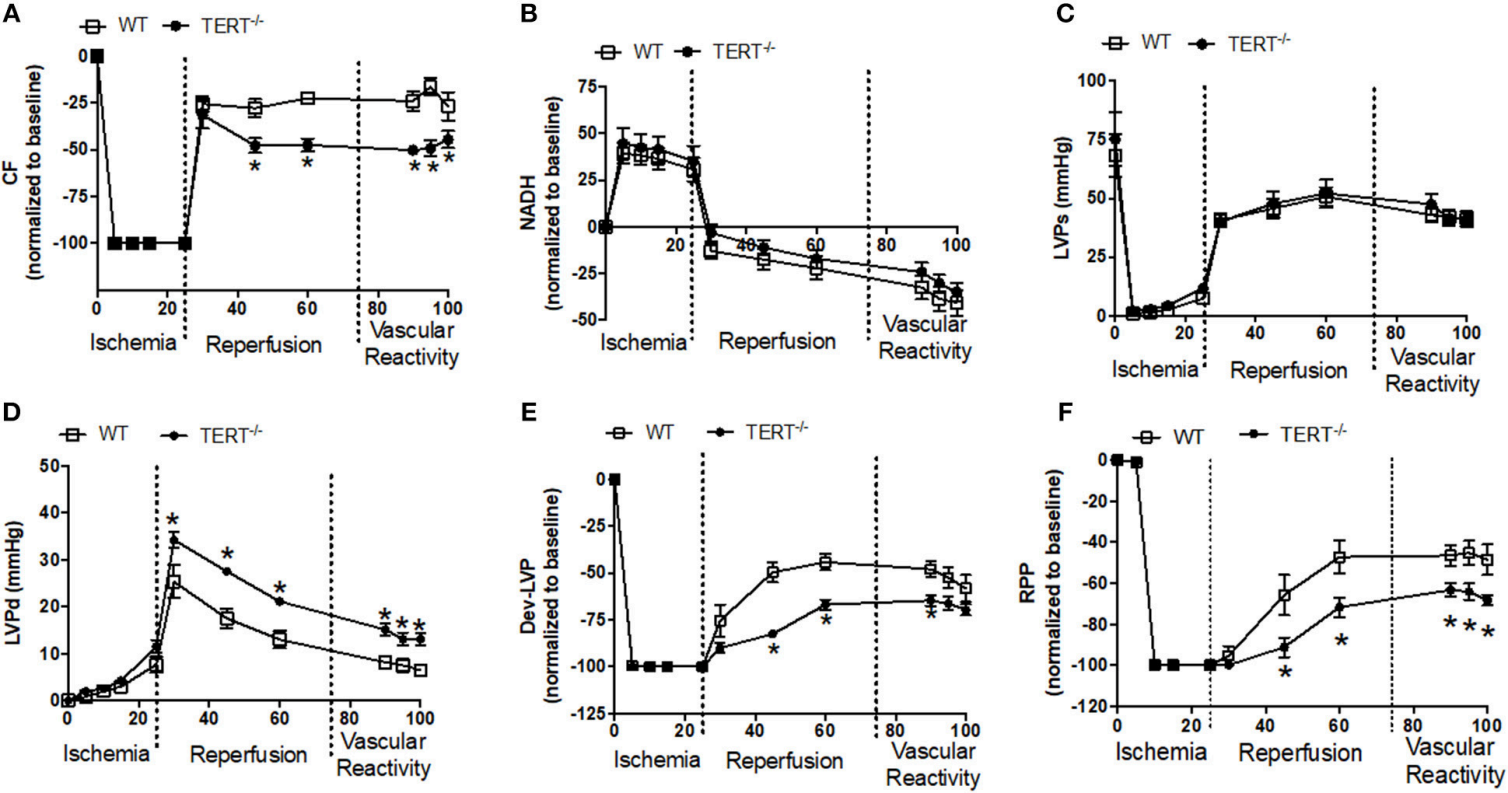
Cardiac function before, during and after global ischemia in TERT^−/−^ and WT without prior Ang II treatment. **(A)** Relative changes in CF; **(B)** Relative changes in NADH autofluorescence; **(C)** Absolute Systolic LVP (LVSP; mmHg); **(D)** Absolute diastolic LVP (LVDP; mmHg); **(E)** Dev- LVP (Difference between LVSP and LVDP) and **(F)** Relative changes in RPP (calculated as LVDP × HR) recorded before, during, and after 25 min no flow, global ischemia for TERT^−/−^ (*n* = 3 males and 5 females) and WT (*n* = 3 males and 4 females) groups. Values are expressed as means ± SEM. *P < 0.05 *t* student test.

Figure 3 summarizes the relative percent (i.e., data normalized to baseline) and absolute changes (mmHg) in cardiovascular functions after I/R in rats untreated with Ang II. Following 25 min global no flow ischemia, TERT^−/−^ isolated hearts showed reduced CF recovery, Dev-LVP and RPP throughout the 120 min reperfusion period compared to WT hearts (Figures 3A,E,F); post-ischemic diastolic LVP was significantly higher in the TERT^−/−^ compared to the WT isolated hearts (Figure 3D). The redox state assessed based on NADH levels and the systolic LVP were not significantly different between TERT KO vs. WT (Figures 3B,C). Vascular responses to BK, SNP, and ADE during late reperfusion were similar between the two groups (Figure 3A), but the relative magnitude of change remained significantly higher in the WT compared to the TERT^−/−^ rats. The similar vascular responses to the vasoactive agents indicate that after the acute I/R, vascular integrity was not compromised by the I/R injury in the TERT^−/−^ and the WT.

**Figure 4:**
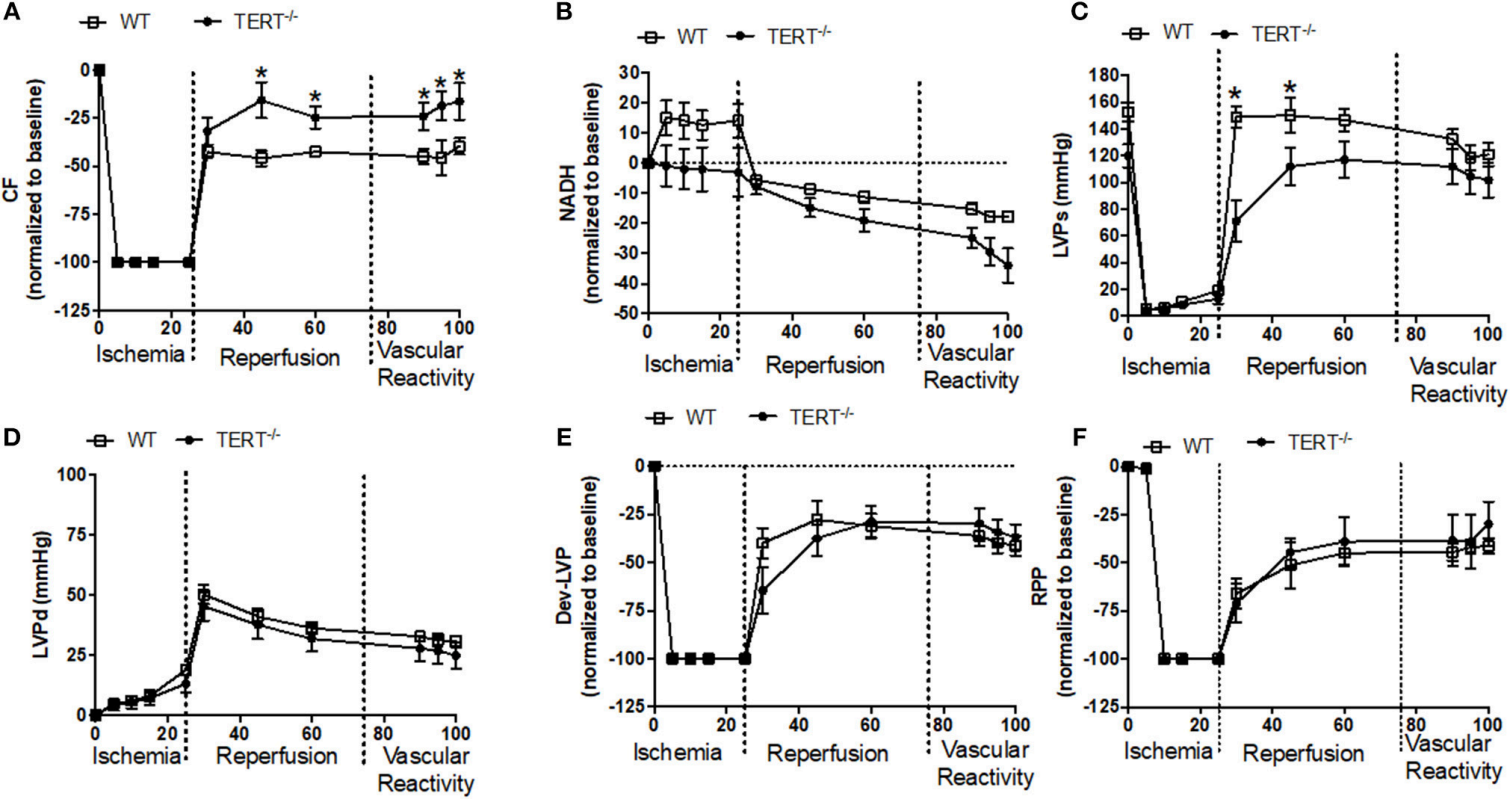
Cardiac function before, during and after global ischemia in TERT^−/−^ and WT after Ang II treatment. **(A)** Relative changes in CF; **(B)** relative changes in NADH autofluorescence; **(C)** absolute Systolic LVP (mmHg); **(D)** absolute diastolic LVP (mmHg); **(E)** relative changes in Dev- LVP; and **(F)** relative changes in RPP recorded before, during, and after 25 min no flow global ischemia for TERT^−/−^ + Ang II (*n* = 2 males and 2 females) and WT + Ang II (*n* = 2 males and 4 females) groups. Values are expressed as means ± SEM. *P < 0.05 *t* student test.

Figure 4 the relative percent (i.e., data normalized to baseline) and absolute changes (mmHg) in cardiovascular functions before, during and after I/R in rats treated with Ang II before IR injury. Post-ischemic diastolic LVP, and RPP were similar between the WT and TERT^−/−^ rats (Figures 4D,F). However, the post-ischemic systolic LVP was lower in the TERT^−/−^ compared to the WT hearts during the first 20 min of reperfusion (Figure 4E). NADH levels decreased (i.e., oxidized) during the ischemia period and continued to decline during the reperfusion period in the TERT^−/−^ compared to WT hearts (Figure 4B). In contrast to the effect of TERT^−/−^ on functional variables during I/R in the Ang II untreated rats (Figure 3A), the TERT^−/−^ hearts displayed higher CF during reperfusion in the Ang II treated hearts compared to the WT hearts (Figure 4A). Despite the difference in CF, the responses to BK SNP and ADE were similar between the two groups. However, the TERT^−/−^ hearts showed significantly greater CF than the WT with the vasoactive agents. The responses to the vasoactive agents demonstrate that the acute I/R injury did not significantly alter the vascular integrity of both groups of rats. It is worth noting that all functional variables and NADH levels remained relatively unchanged throughout the perfusion period in the time control (data not shown) group, which indicates stability and reliability of the Langendorff IR procedure.

### Absence of Telomerase Promotes _mt_DNA Lesion and Mitochondrial Dysfunction

**Figure 5:**
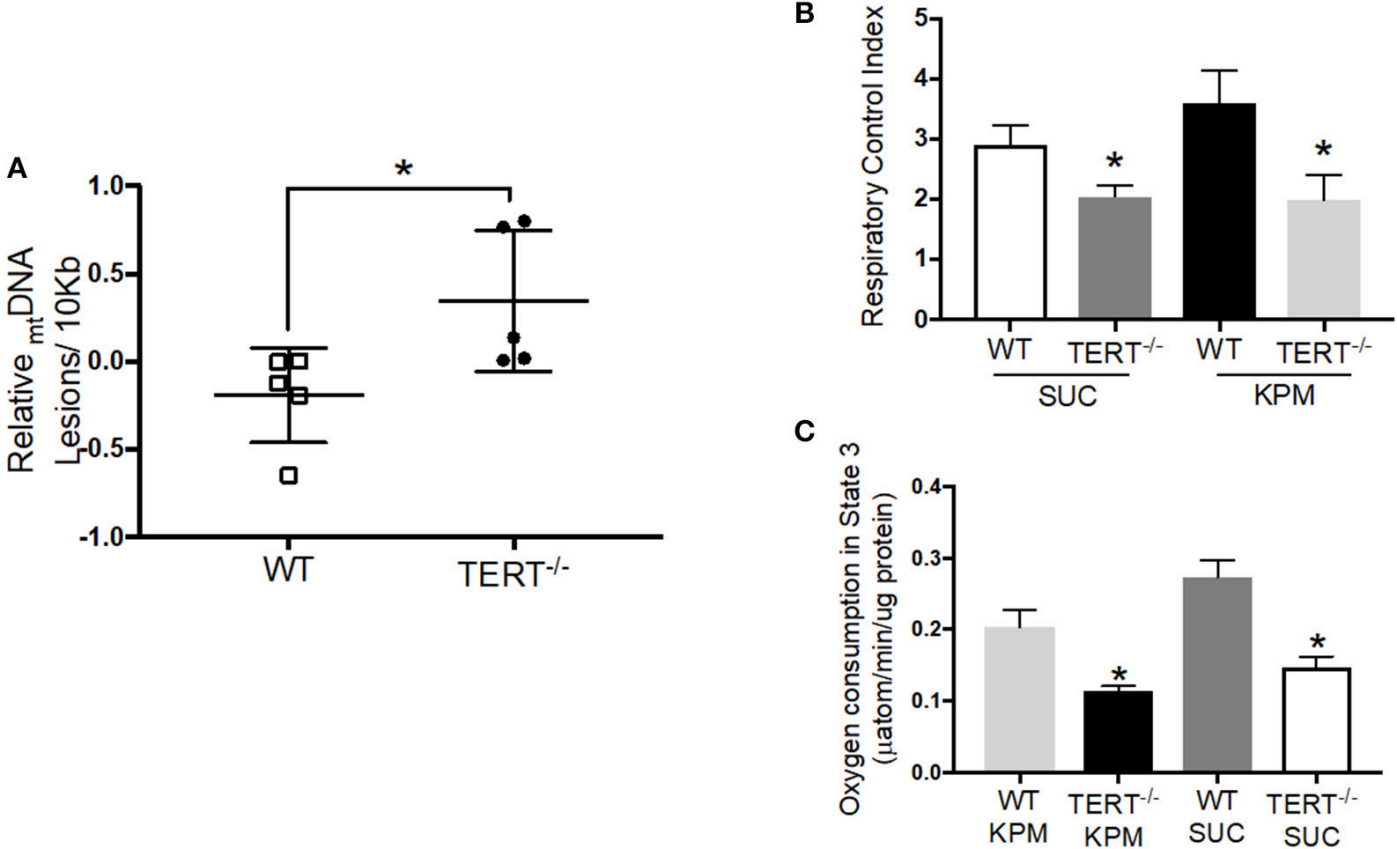
_mt_DNA integrity and mitochondrial respiration in TERT^−/−^ and WT rat hearts not treated with Ang II. **(A)**_mt_DNA lesions level measured in _mt_DNA isolated from rat hearts; *n* = 5 in each group; *p = 0.04 for hearts from TERT^−/−^ rats vs. hearts from WT. **(B)** Respiratory Control Index (RCI) of isolated mitochondria of fresh rat hearts in the presence of potassium pyruvate-malate (KPM) or Succinate (SUC); *n* = 4–5; *p = 0.043 and *p* = 0.042 for mitochondria from TERT^−/−^ vs. WT hearts with SUC and KPM respectively. **(C)** Oxygen consumption in State 3 of isolated mitochondria of fresh rat hearts in the presence of potassium pyruvate-malate (KPM) or Succinate (SUC); *n* = 4–5; *p = 0.0001 and *p* = 0.004 for mitochondria from TERT^−/−^ vs. WT hearts with SUC and KPM, respectively. Values are expressed as mean ± SEM; *P < 0.05 *t* student test and One-way ANOVA.

Telomerase plays a major role in protecting against oxidative damage and _mt_DNA damage, and thereby preserve or enhance the respiratory chain activity (27). Based on these observations, we sought to evaluate the effect of the genetic absence of telomerase activity on mitochondrial integrity. To directly assess _mt_DNA integrity, total genomic DNA was isolated from total hearts of TERT^−/−^ and WT rats to perform PCR-based assay of damaged _mt_DNA. Mitochondrial DNA lesions increased significantly in hearts from untreated TERT^−/−^ compared to WT (*N* = 5; *p* < 0.05) (Figure 5A).

### Mitochondrial Respiration

To directly assess mitochondrial respiratory capacity and the effectiveness of OXPHOS to convert the added ADP to ATP, we measured O_2_ consumption in mitochondria isolated from fresh hearts of TERT^−/−^ and WT rats using the Clark-type oxygraph. Figures 5B,C show RCIs and O_2_ consumption rates in state 3, respectively, during oxidation of the complex I substrate potassium-pyruvate/malate (KPM), and the complex II substrate succinate (SUC). The RCIs and the O_2_ consumption during state 3 respiration were significantly lower in mitochondria isolated from TERT^−/−^ hearts compared to WT control hearts, without Ang II treatment (KPM-RCI: WT 3.6 ± 0.5 and TERT^−/−^ 1.9 ± 0.4^*^; SUC-RCI: WT 2.9 ± 0.3, and TERT^−/−^ 2.1 ± 0.2^*^, *n* = 4–5; ^*^p < 0.05 for TERT^−/−^ vs. WT).

## Discussion

Major findings of this study are summarized as follows: (1) Genetic disruption of the TERT gene did not alter basal *in vivo* nor *ex vivo* cardiac function; however, after Ang II treatment, *ex vivo* basal LVP was decreased in KO rats. (2) Paradoxically, infarct size was less in the TERT KO hearts compared to the WT. (3) Contrary to the infarct size, deficiency in telomerase activity was associated with significant decrease in post-ischemic CF, Dev-LVP, and RPP. (4) Pretreatment with Ang II annulled the post-ischemic differences observed between the TERT^−/−^ and WT rats, but exacerbated infarct size in both WT and in TERT^−/−^; however, in TERT^−/−^ rats the effect of Ang II on infarct size was significantly augmented. (5) Lack of telomerase activity increased _mt_DNA lesion and decreased OXPHOS respiratory capacity (RCI) during oxidation of complexes I and II substrates in mitochondria isolated from hearts.

Telomerase is an enzyme complex responsible for maintaining telomere length (56). It consists of two major components, the catalytic subunit TERT and the RNA-template TERC (57). With aging, telomerase activity decreases and telomeres shorten in all cells during division (58). Evidence shows a positive correlation between telomere shortening, decreased telomerase activity and heart disease (6, 59–61). In addition, a recent large cohort study demonstrated a strong association between shorter telomeres and higher risk of ischemic heart disease (62). However, as most cells in the heart (myocytes and endothelial cells) undergo very low rates of cell division, a physiological role of non-nuclear telomerase seems logical. In the present study, the TERT^−/−^ rats showed loss of telomerase activity and a subsequent significant telomere shortening. However, this telomere shortening is not considered critical (critical short telomeres < 1,000bp). Insofar as rodents have 10–100 times longer telomeres than humans, the observed shortening in our rats should not result in chromosomal rearrangement. Supporting this notion is the fact that in mice lacking telomerase activity (either TERT or TERC knock outs) display a telomere loss at a rate of ~5 Kb per generation and no dramatic loss of viability until the 3rd and 4th generation (63). Since the goal of the present study was to evaluate the effect of the loss of telomerase activity on susceptibility to I/R injury without critical short telomeres, the 1st generation of the knockout rats constitute the ideal model for this study.

I/R injury is often part of several clinical events such as cardiac arrest and resuscitation, and coronary artery occlusion and reperfusion. Most of the injury occurs during the early phase of reperfusion (64), which may be attributed mainly to excess ROS emission, Ca^2+^ overload, and concomitantly, induction of apoptosis (65). Although the TERT^−/−^ and the WT counterparts displayed similar cardiac function and structure as shown by echocardiography and *ex vivo* pre-ischemia cardiac analysis, basal LVP (Table 1) in the TERT^−/−^ I/R hearts from the Ang II treated group exhibited reduced Dev-LVP and RPP, and higher diastolic LVP. During post-ischemia, cardiac dysfunction and cell death are mediated, in part, by factors that facilitate cell death. Even though in the Ang II untreated rats the infarct size was similar in the TERT^−/−^ hearts compared to the WT, the strong recovery of LVP in the WT suggests that other factors, possibly, mitochondrial function may be involved in functional recovery after I/R.

In response to reperfusion, the restore of flow introduces O_2_ to the ischemic heart that leads to burst of further ROS production (65). Reports have shown increased ROS impairs cardiomyocyte calcium channels and thereby intracellular Ca^2+^ homeostasis (66). Cytosolic Ca^2+^ dysregulation could also be attributed to profound decline in mitochondria ability to generate ATP, necessary for regulation of cytosolic Ca^2+^. Impaired ATP production contributes to net Ca^2+^ accumulation and ROS production in cardiomyocytes, which may lead to cell death (67). We have shown in numerous studies that I/R in the *ex vivo* model leads to increase cytosolic and mitochondrial Ca^2+^ overload and ROS emission (46–48). These changes contribute to significant attenuation of cardiac function and increase infarction during reperfusion. Although we did not measure these parameters in our current study, it is highly likely that these are contributing factors in the compromise of cardiac function on reperfusion (Figures 3A,E,F). Therefore, the worsening in function in the TERT^−/−^ could be attributed to derangement in mitochondrial Ca^2+^ homeostasis and ROS production, which leads to compromised ATP production and contractile dysfunction.

The underlying cellular mechanisms how lack of TERT affects cardiac function remain to be explored. Based on existing knowledge, we know that increased levels of intracellular Ca^2+^ trigger cellular senescence (68, 69). Consistent with our stated hypothesis above, several studies have demonstrated that pharmacological inhibition of telomerase induces cellular senescence (70–72). Altogether, this suggests that in the absence of telomerase, the heart is more prone to senescence in response to higher oxidative stress and increased intracellular Ca^2+^ during I/R injury. Surprisingly, and unexpectedly, TERT KO resulted in lesser infarction when compared to the WT in the Ang II untreated rat hearts (73, 74). This is in contrast to previous work by Bär et al. who showed that acute overexpression of TERT in mouse models of myocardial infarct improves survival and decreases infarct size after I/R injury (7). It is well known that telomerase plays an important role in apoptosis (75). However, Santos et al. have shown that depending on its localization (mitochondria vs. nucleus), the catalytic subunit TERT is able to render the cell more susceptible to apoptosis (24). This suggests that in a global KO system such as in our TERT^−/−^ rat model, TERT was certainly not able to prevent the functional damage following I/R (oxidative stress and mitochondrial damage), but it was also less susceptible to apoptosis (which involves TERT's localization to the nucleus) and consequently equal infarct scars. Pre-treatment of rats with Ang II negated these differences in infarction and resulted in higher infarct size in both groups, but more so in the TERT^−/−^. Despite the larger infarct size in the TERT^−/−^ there was no significant difference in between the two groups in most of the functional variables, except in coronary flow, which was significantly higher in the Ang II treated hearts (Figure 4). This suggests that telomerase KO has yet, undefined role in the *ex vivo* cardiac IR injury model. Further studies are required to unravel the underlying mechanisms that contribute to these events. Mitochondrial link to this process could be a potential avenue for further investigation, as we have attempted in this study.

Thus, beside its telomeric function in the nucleus, it is now well known that the catalytic subunit TERT can translocate to mitochondria where it has been reported to play a role in cyto-protection against oxidative stress (6, 24, 26). In cardiomyocytes, mitochondria are the primary source of ROS and they are also more susceptible to oxidative damage. During reperfusion, the major source of ROS in cardiomyocytes is the electron transport chain (ETC), which is gradually damaged during a vicious cycle of ROS-induced-ROS release during I/R (76–81). Mitochondrial complex I, a key component of the ETC, aerobically oxidizes NADH in the ETC to generate ATP. In the absence of oxygen (ischemia), NADH accumulates (highly reduced) because complex IV is blocked; in the current study, as it has been shown previously (48, 82), NADH increased (reduced redox state) during ischemia, but to the same levels in the TERT^−/−^ and WT rats; on reperfusion, the mitochondria were relatively more reduced in the WT compared to the TERT^−/−^ in the Ang II untreated rats, which would suggest better availability of reducing equivalents (NADH/FADH_2_) for OXPHOS to provide ATP for better recovery of LVP and RPP in the WT. However, in the hearts treated with Ang II, the reduced and oxidized states (redox) of NADH during I/R were not different between TERT^−/−^ and WT. The similar redox states in the two groups during I/R would portend similar OXPHOS and ATP production that results in similar functional recovery (e.g., LVP and RPP).

Unlike the nuclear DNA protected by histones and other nuclear factors (e.g., introns), _mt_DNA are particularly sensitive to oxidative stress due to their proximity to the ETC and the lack of histones (83). Much of the _mt_DNA is used to code the manufacture of proteins that are key components of the energy production system, including subunits of some of the ETC complexes. TERT is known to bind with the _mt_DNA regions encoding some complex I (NADH-ubiquinone oxidoreductase) subunits, which may enhance their activity (27) and potentially increase OXPHOS. Our data shows TERT^−/−^ rats display a higher level of _mt_DNA damage accompanied by a significant decrease in mitochondrial coupling of OXPHOS. Taken together, these data suggest that in the absence of TERT translocation to mitochondria, _mt_DNA and ETC become more susceptible to oxidative stress-mediated damage that could promote cardiac defects observed in our findings. Furthermore, in the absence of Ang II, these rats show significant deficit in functional recovery on reperfusion after global ischemia.

### Study Limitations

There are several limitations that need to be taken into consideration in this study. First, with the experimental design that has been chosen to generate the KO rats, a variant of the protein TERT was created, as detected by the antibody used. Although, the activity assay confirmed its non-telomeric function, we cannot exclude the possibility that this variant might have a different unknown function. Furthermore, studies of mass spectrometry and function are required to establish its sequence and potential function.

Second, following the ablation of telomerase activity, a significant telomere shortening was observed in the TERT^−/−^ rats. While this does not affect the life-span of these rats at the first generation (63), we can only speculate that this shortening is not critical for other cellular functions (e.g., proliferation, migration…etc.). Our previous studies suggest a non-canonical role of telomerase in the vascular bed and mitochondria (6, 30). Separating these two actions of TERT could help delineate the effect of telomere shortening vs. TERT loss.

We have used Ang II as a systemic stressor for this study because of its widely-reported effect of oxidative damage and induction of cardiovascular dysfunction (84, 85). However, Ang II does not act only locally but rather affects most organ systems including the kidney, sympathetic nervous system, and vascular function that contribute to the regulation of systemic blood pressure, cardiac function and other physiological processes (86). Due to these systemic effects of Ang II, we cannot exclude that the observed phenotype is direct effect of Ang II on cardiac function or subsequent to other organ defects.

## Conclusions

This study demonstrates that functional deletion of TERT predisposes to pathological changes of the heart in function and structure that are consistent with signs of heart failure and decrease functional recovery after an I/R event. Our findings confirm that TERT plays a crucial role regulating mitochondrial functions that are critical in the recovery of cardiac function after an ischemic event.

## Author Contributions

KA-A, AC and AB: conception and design, drafting and revising of manuscript, critical review, final approval of the manuscript submitted. JH, LN, LH, GD and DB: experimentation and data analysis. AG: animal model generation.

### Conflict of Interest Statement

The authors declare that the research was conducted in the absence of any commercial or financial relationships that could be construed as a potential conflict of interest.

## Supplementary Material

Supplemental Figure 1 Summary of baseline and after Ang II infusion echocardiogram characteristics of TERT^−/−^ and WT rats. **(A)** Left ventricle dimensions: Interventricular septal (IVS); Left ventricular internal diameter (LVID; s, systole and d, diastole); Left ventricular posterior wall thickness at end-diastole (LVPWd); **(B)** END-diastolic volume (EDV); End-systolic volume (ESV) and Stroke volume (SV); **(C)** left ventricular mass (LVM); **(D)** Heart rate (HR) recorded in TERT^−/−^ (*n* = 6 males and 7 females) and WT (*n* = 6 males and 6 females) groups before or after Ang II (TERT^−/−^: *n* = 2 males and 2 females and WT: *n* = 2 males and 4 females). Values are expressed as mean ± SEM.

Supplemental Figure 2 A frame shifting indel in exon 1 eliminates TERT expression in WKY rats. **(A)** Left—A 17-bp deletion was induced in the first exon of the WKY TERT gene using CRISPR/Cas9 and confirmed by Sanger sequencing. **(B)** Validation of the CRISPR targeting whole TERT by genotyping.
