# Supplementary figures and images for: Telomerase Deficiency Predisposes to Heart Failure and Ischemia-Reperfusion Injury
Source: Front Cardiovasc Med. 2019 Apr 2;6:31. doi: 10.3389/fcvm.2019.00031 (PMC6454001; doi:10.3389/fcvm.2019.00031)

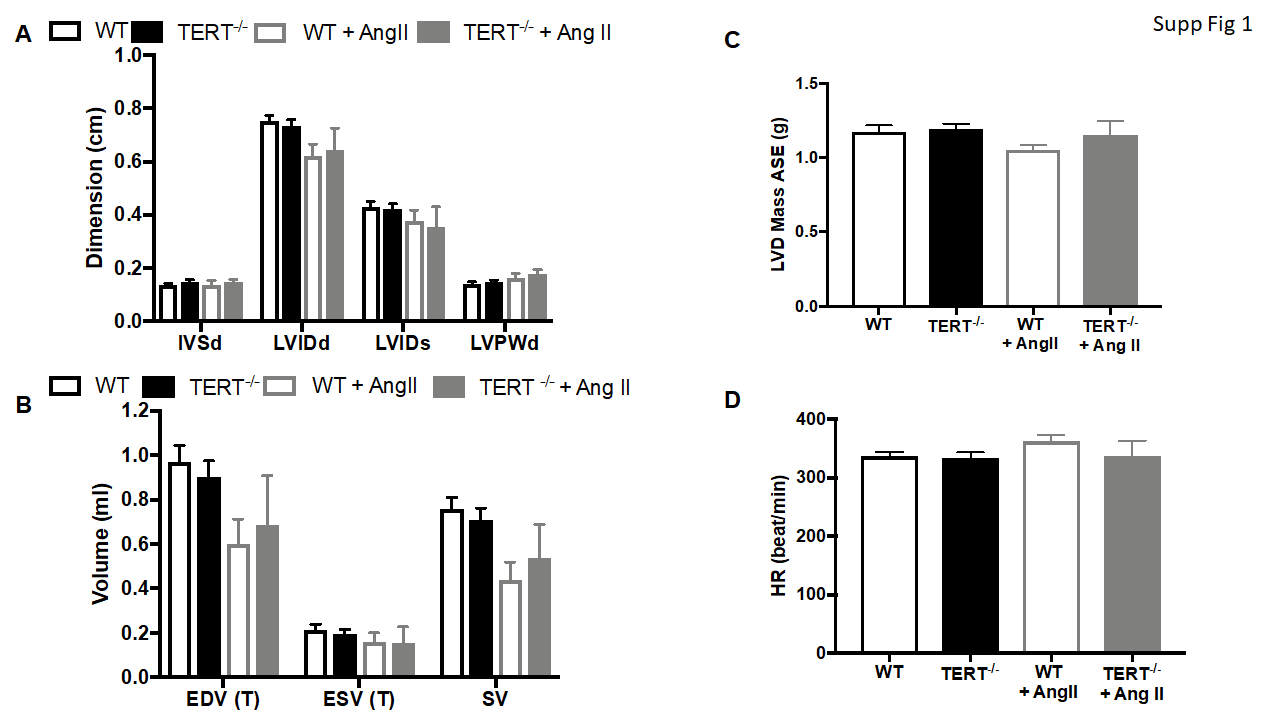

Supplement: Supplemental Figure 1 — Summary of baseline and after Ang II infusion echocardiogram characteristics of TERT−/− and WT rats. (A) Left ventricle dimensions: Interventricular septal (IVS); Left ventricular internal diameter (LVID; s, systole and d, diastole); Left ventricular posterior wall thickness at end-diastole (LVPWd); (B) END-diastolic volume (EDV); End-systolic volume (ESV) and Stroke volume (SV); (C) left ventricular mass (LVM); (D) Heart rate (HR) recorded in TERT−/− (n = 6 males and 7 females) and WT (n = 6 males and 6 females) groups before or after Ang II (TERT−/−: n = 2 males and 2 females and WT: n = 2 males and 4 females). Values are expressed as mean ± SEM. [file Image_1.tif]

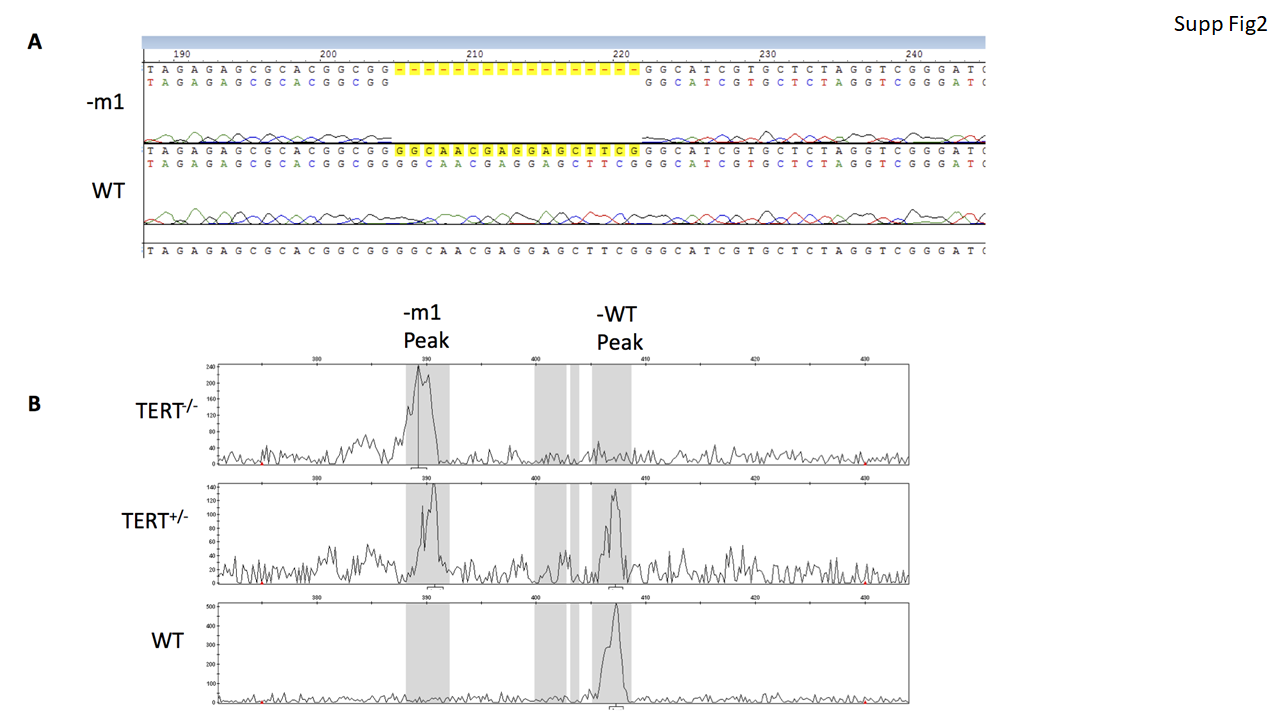

Supplement: Supplemental Figure 2 — A frame shifting indel in exon 1 eliminates TERT expression in WKY rats. (A) Left—A 17-bp deletion was induced in the first exon of the WKY TERT gene using CRISPR/Cas9 and confirmed by Sanger sequencing. (B) Validation of the CRISPR targeting whole TERT by genotyping. [file Image_2.tif]
